# Supplementary material for: Statistical potentials from the Gaussian scaling behaviour of chain fragments buried within protein globules
Source: PLoS One. 2022 Jan 27;17(1):e0254969. doi: 10.1371/journal.pone.0254969 (PMC8794220; doi:10.1371/journal.pone.0254969)
Supplement: S1 File — (PDF) [file pone.0254969.s001.pdf]

Supplementary material for

Statistical potentials from the Gaussian scaling behaviour of chain fragments buried within protein globules

Stefano Zamuner[1], Flavio Seno[2,3] and Antonio Trovato[2,3] *

[1] Institute of Physics, École Polytechnique Fédérale de Lausanne, CH-1015 Lausanne, Switzerland

[2] Department of Physics and Astronomy, University of Padova, Via Marzolo 8, I-35131 Padova, Italy

[3] INFN, Sezione di Padova, Via Marzolo 8, I-35131 Padova, Italy

* Correspondence to: antonio.trovato@unipd.it

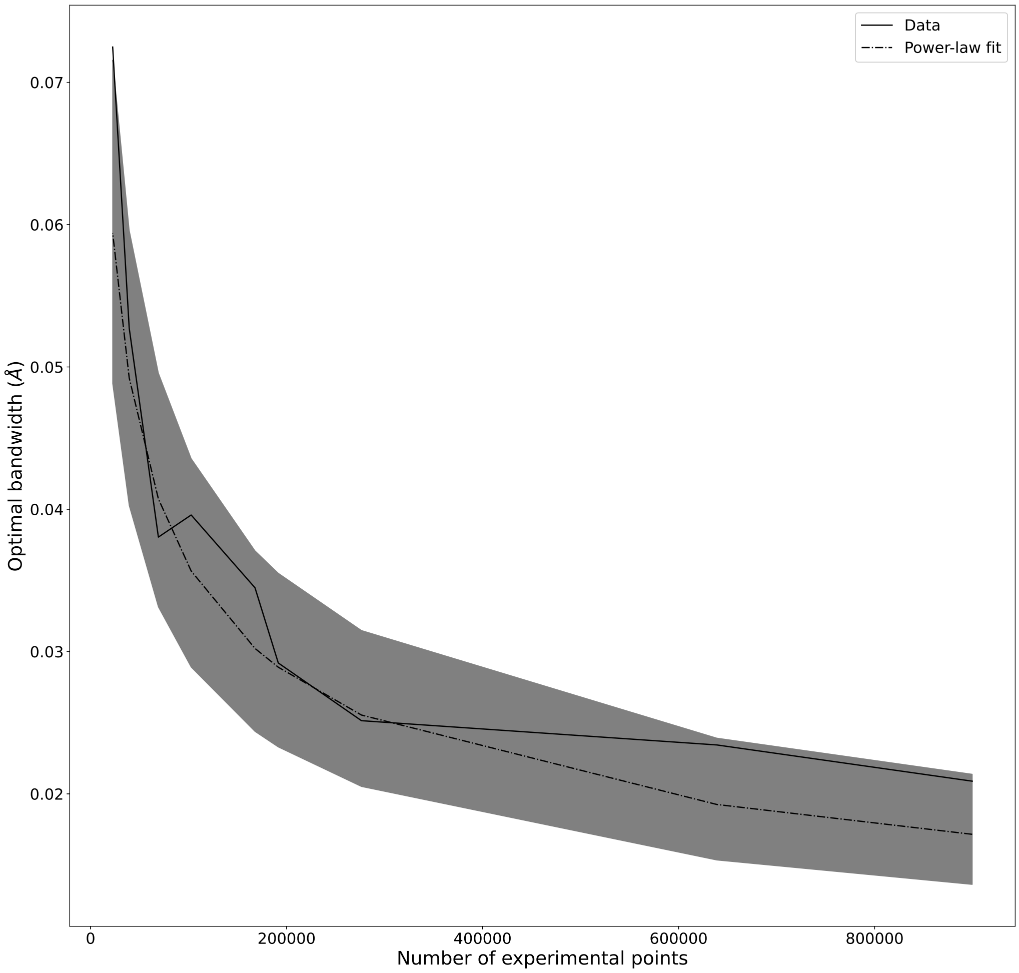

FIG. S1. **Optimal KDE bandwith vs sample size.** Optimal KDE bandwidth w as a function of empirical sample size n. The optimal bandwith was determined for the subset of fragment lengths $\{42, 48, 60, 64, 66, 72, 78, 84, 92\}$ by maximizing the likelihood to the empirical distribution with a cross validation procedure. The dashed line is the powerlaw fit $w(n) = an^s$, with a, s determined by minimizing the RMSD with the cross-validated bandwidths.

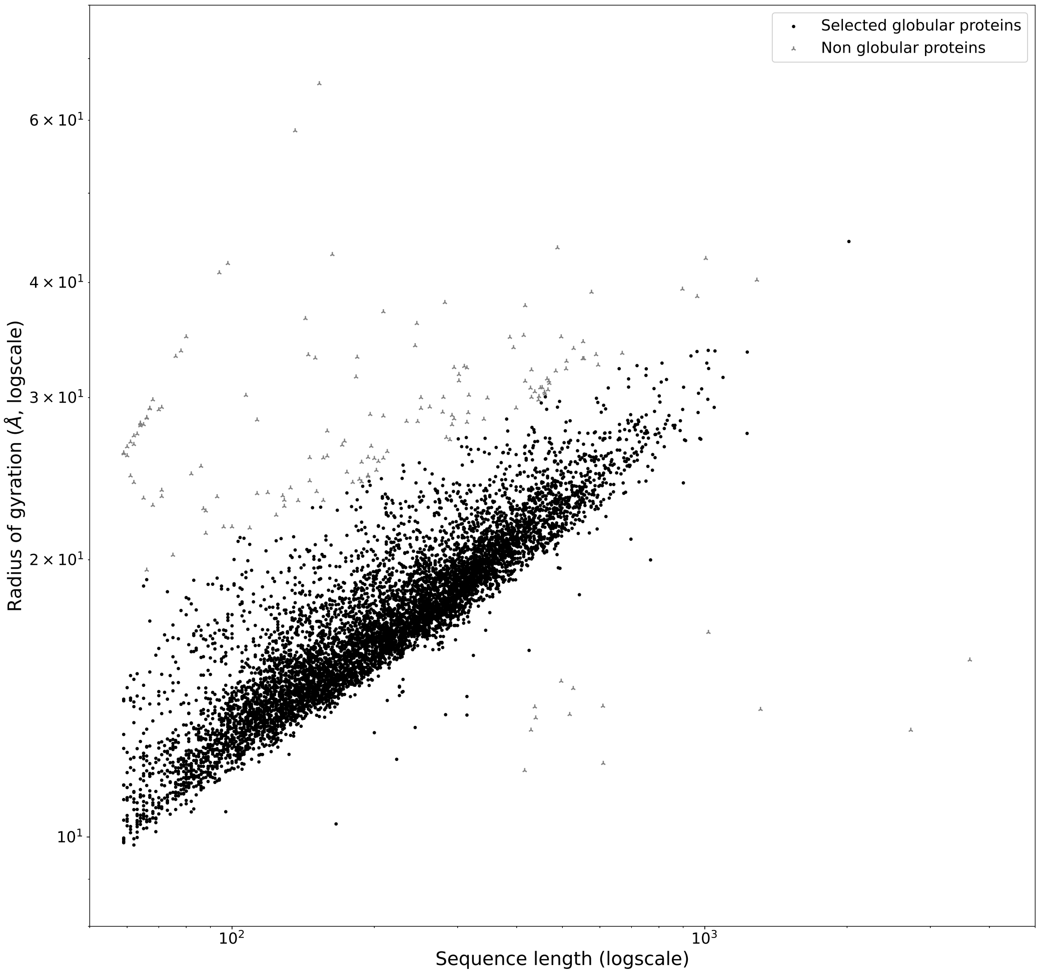

FIG. S2. **Data set pruning: gyration radius vs length.** Scatter plot of gyration radius vs sequence length for all proteins in the Top8000 data set (http://kinemage.biochem.duke.edu/databases/top8000.php) Full circles denote the proteins selected for subsequent analysis in our work. Grey symbols denote the 164 proteins that were discarded since they fall more than three standard deviations apart from the power law $R_g(N) = aN^{1/3}$ fitted to all data.

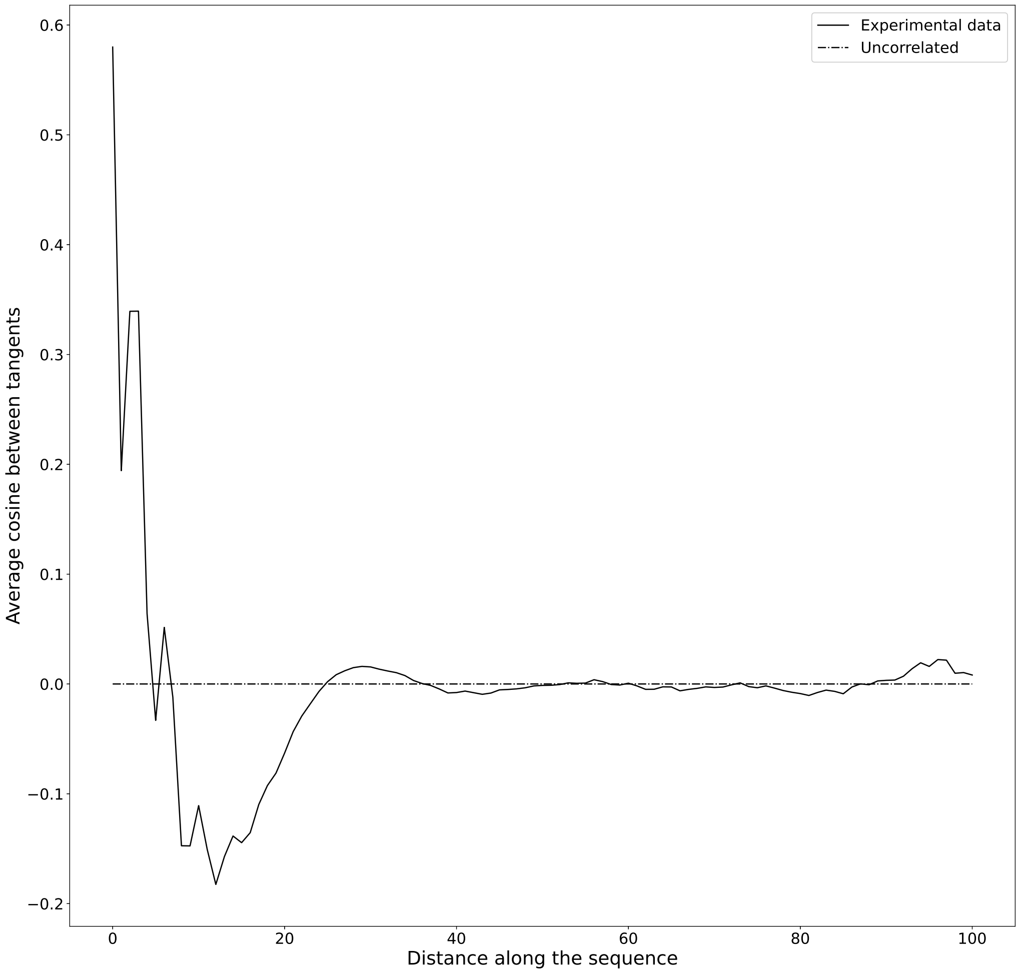

FIG. S3. **Tangent-tangent correlation function.** Tangent-tangent correlation as a function of sequence separation along the protein chain. Average over all fragments of lengths m satisfying the constraint $m < N^{2/3}$ used to select only fragments buried within the protein globule (N is the length of the whole protein chain).

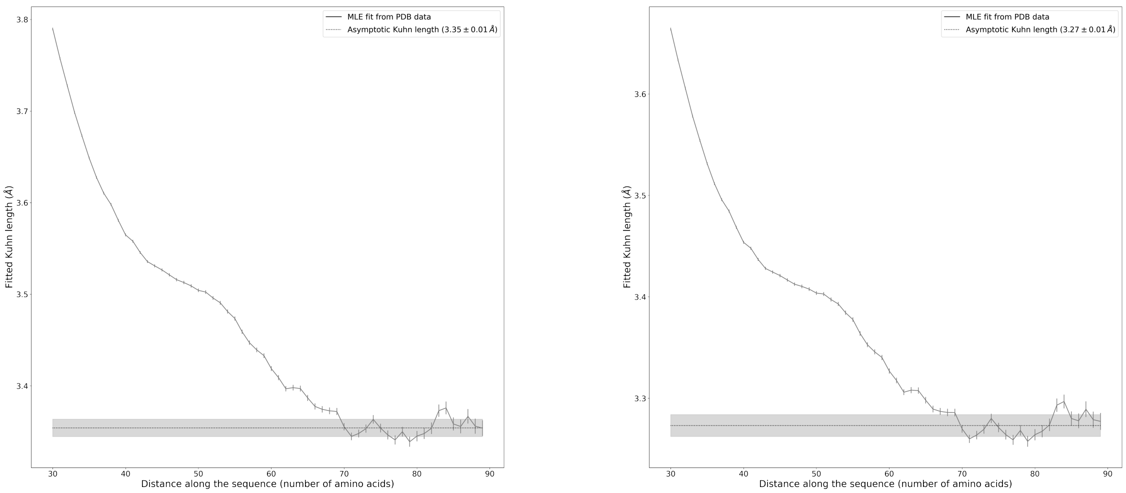

FIG. S4. **Kuhn length vs fragment length.** The Kuhn length $b(m)$, obtained by maximizing the likelihood of the empirical end-to-end distance data to the Maxwell distribution, plotted versus the length m of the protein fragments considered in the statistical analysis. **Left panel**: HH representation (all atoms, including hydrogen atoms). **Right panel**: HV representation (all heavy atoms, excluding hydrogen atoms). The error bars were estimated based on the Fisher information evaluated at $b(m)$ (see main text). For both coarse-graining levels, the values of b decrease monotonically and reach a plateau in the region $70 \leq m \leq 90$. The plateau uniform value is estimated to be $b^* = 3.27 \pm 0.01$ Å for HH and $b^* = 3.35 \pm 0.01$ Å for HV.
.

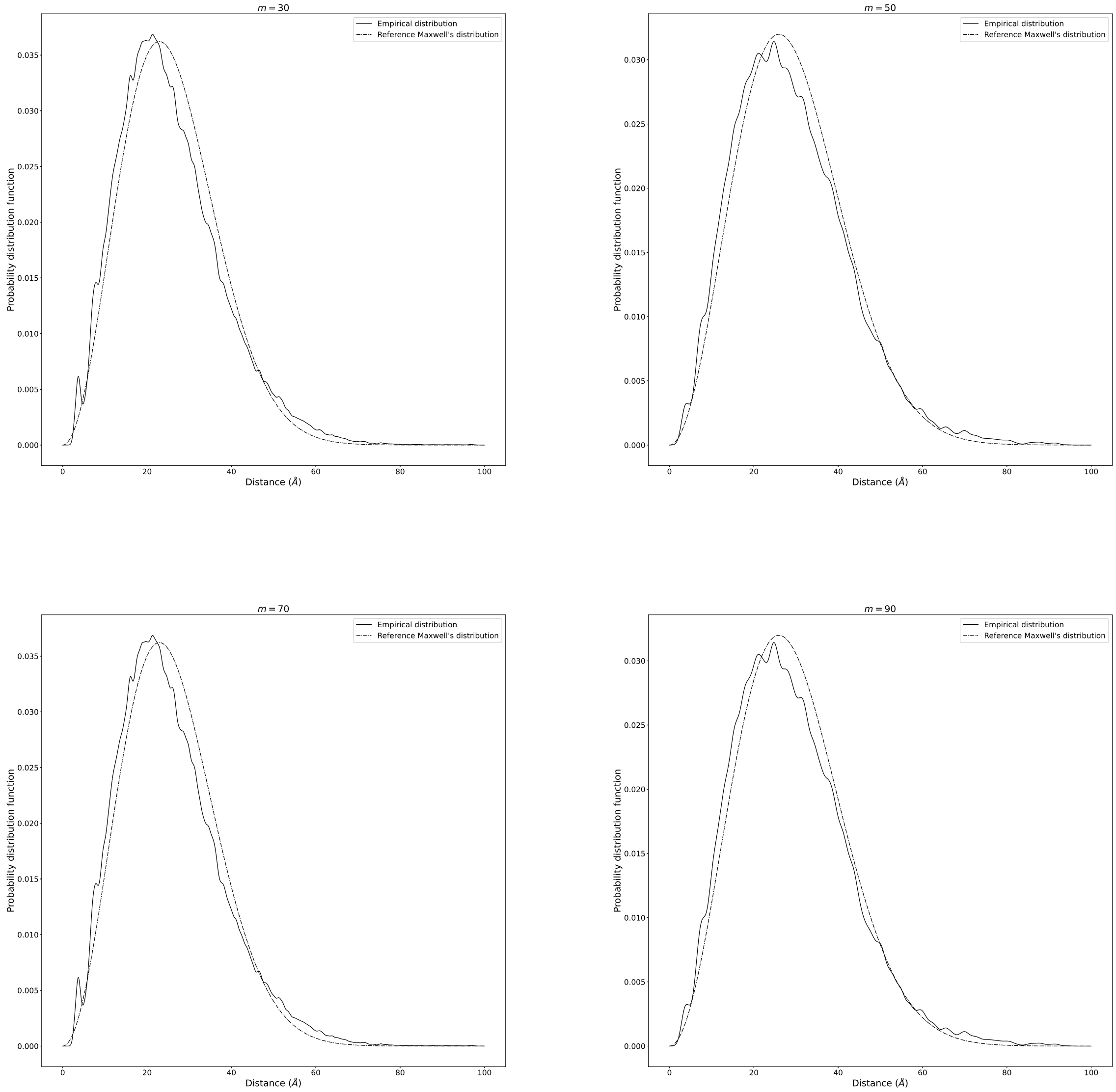

FIG. S5. **End-to-end distance distributions, HV representation.** End-to-end distance probability distributions in the HV representation (all heavy atoms, excluding hydrogen atoms) for four different fragment lengths are shown together with their best fits to Maxwell distributions. The parameters b used in the plot are obtained maximizing the likelihood that the empirical data follow the Maxwell distribution. **Top left panel:** $m = 30$. **Top right panel:** $m = 50$. **Bottom left panel:** $m = 70$. **Bottom right panel:** $m = 90$.

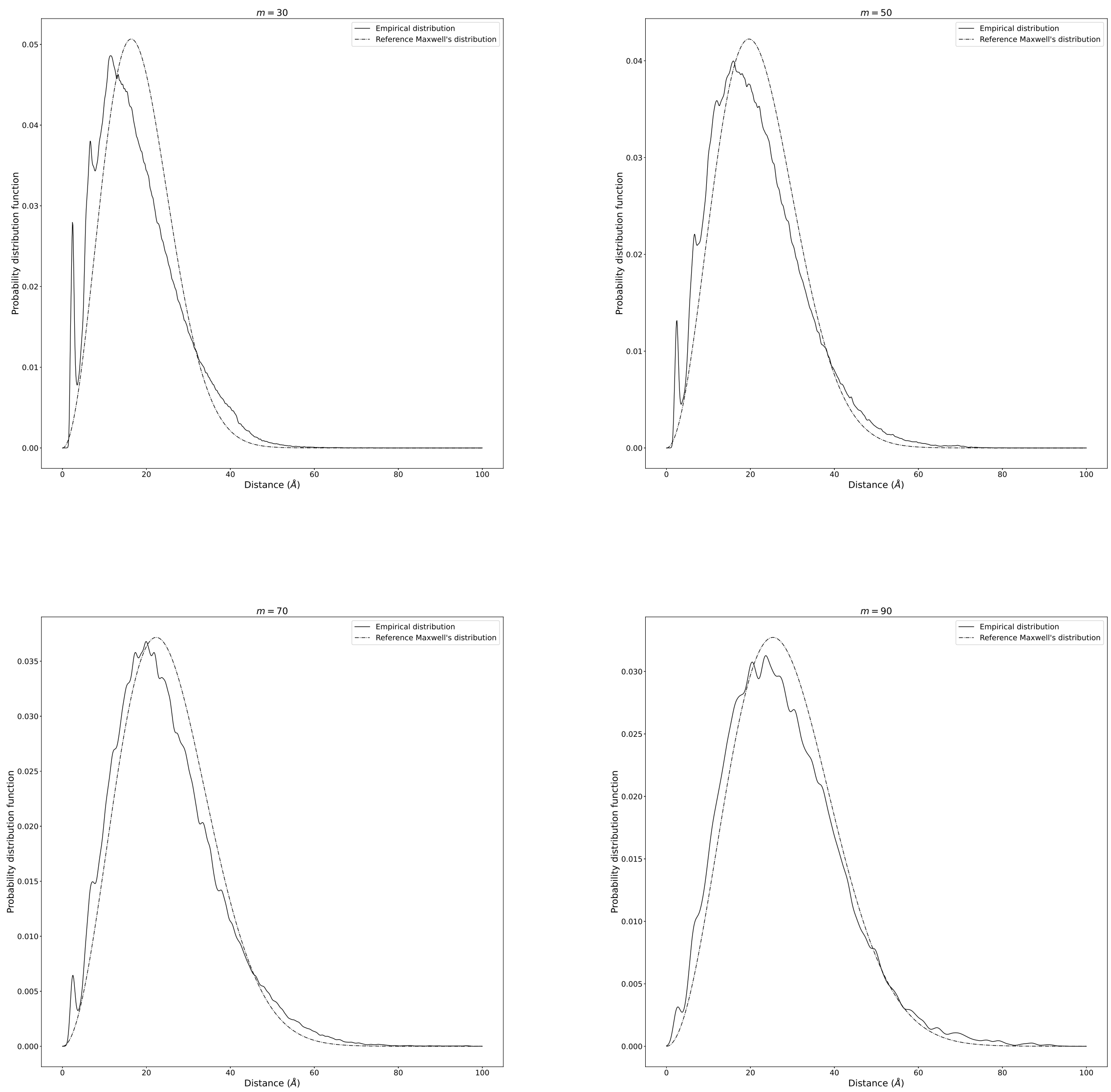

FIG. S6. **End-to-end distance distributions, HH representation.** End-to-end distance probability distributions in the HH representation (all heavy atoms, including hydrogen atoms) for four different fragment lengths are shown together with their best fits to Maxwell distributions. The parameters b used in the plot are obtained maximizing the likelihood that the empirical data follow the Maxwell distribution. **Top left panel:** $m = 30$. **Top right panel:** $m = 50$. **Bottom left panel:** $m = 70$. **Bottom right panel:** $m = 90$.

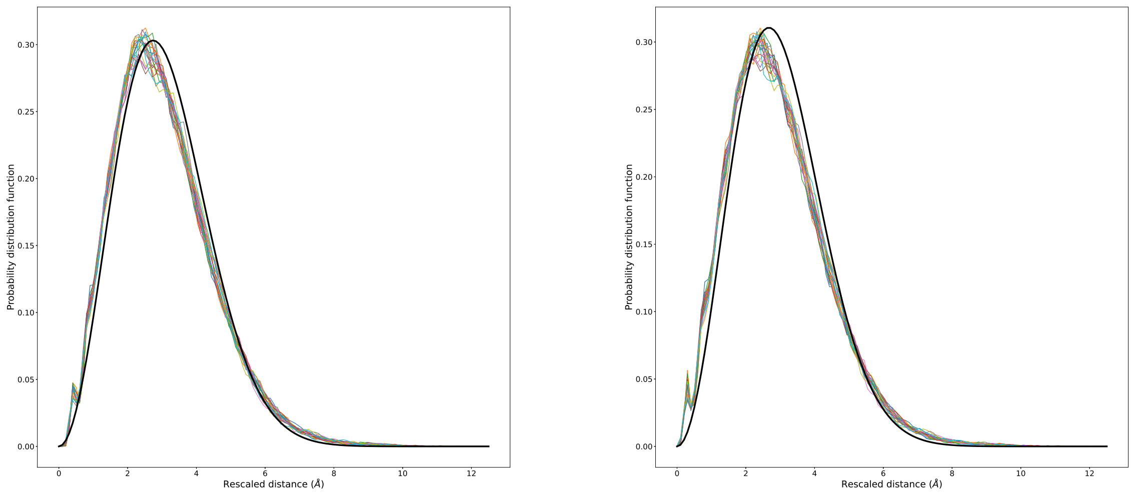

FIG. S7. **Rescaled end-to-end distance distributions collapse in the Flory regime.** The rescaled empirical probability distribution as a function of the rescaled length $R/m^{1/2}$ for $70 \leq m \leq 90$. All curves collapse rather well together and the agreement with the Maxwell distribution with scale parameter b^* (solid black line), determined as the plateaux uniform value, is remarkable. **Left panel:** HV representation. **Right panel:** HH represenation.

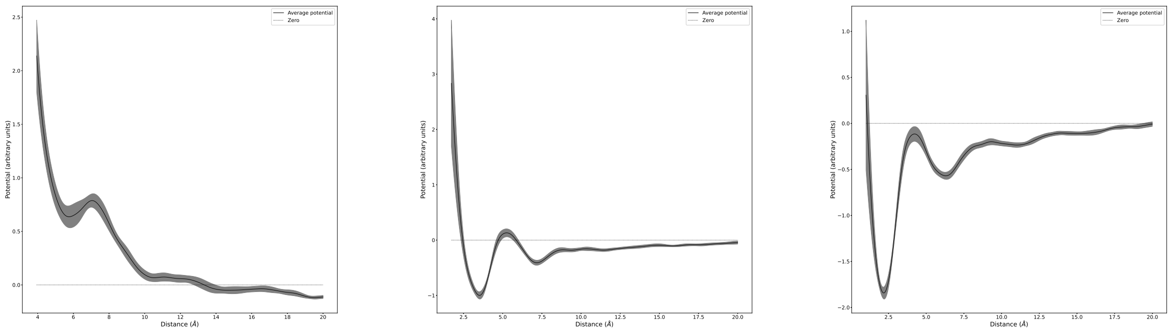

FIG. S8. **Average effective statistical potential in the Flory regime.** The statistical effective potential $V^*(R)$ obtained as the average over the different values of the sequence separation $70 \leq m \leq 90$ in the Flory regime. The corresponding standard deviation is also shown. The Maxwell distribution with effectively uniform Kuhn length $b(m) \simeq b^*$ is used as the ideal reference state. **Left panel:** CA representation. **Middle panel:** HV representation. **Right panel:** HH representation.

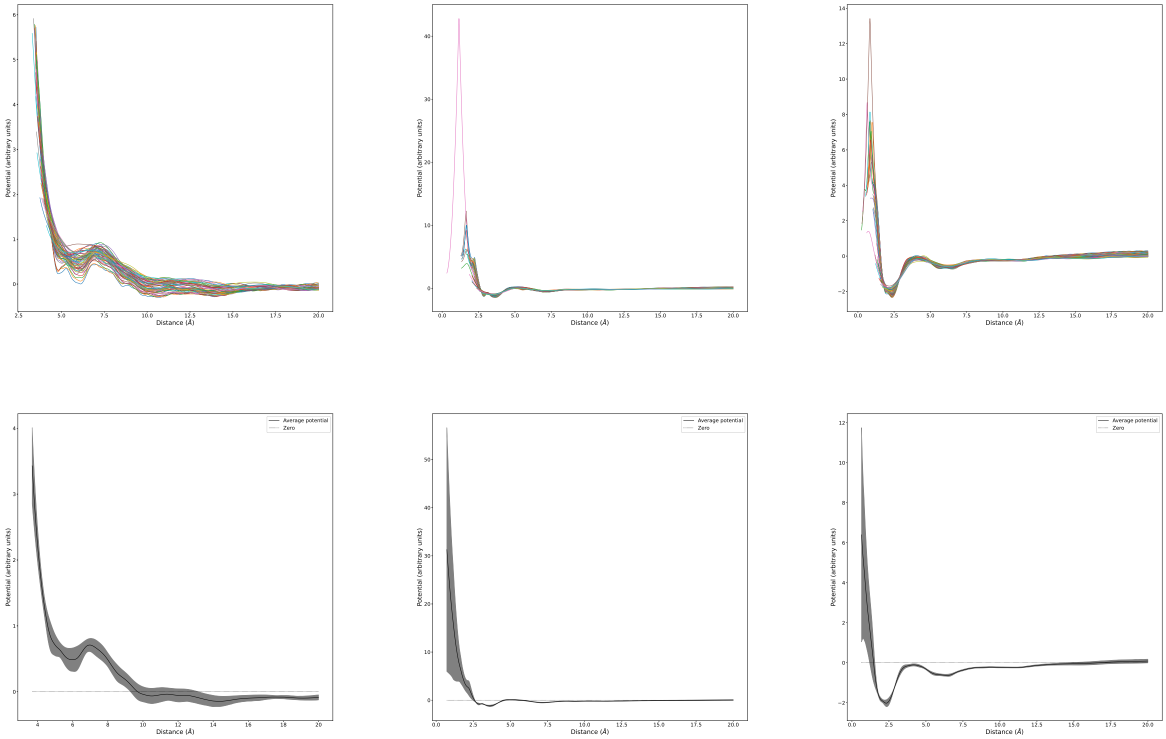

FIG. S9. **Average effective statistical potential with varying Kuhn length.** Data collapse of the effective potential $V_m(R)$ estimated for different values of the sequence separation using the Maxwell distribution with varying Kuhn length $b(M)$ as the ideal reference state. **Top panels:** all curves are shown separately. **Bottom panels:** the average potential $\overline{V}(R)$ with the corresponding standard deviation. **Left panels:** CA representation; all sequence separations $30 \leq m \leq 90$ are used. **Middle panels:** HV representation; all sequence separations $30 \leq m \leq 90$ are used. **Right panels:** HH representation; all sequence separations $30 \leq m \leq 90$ are used.

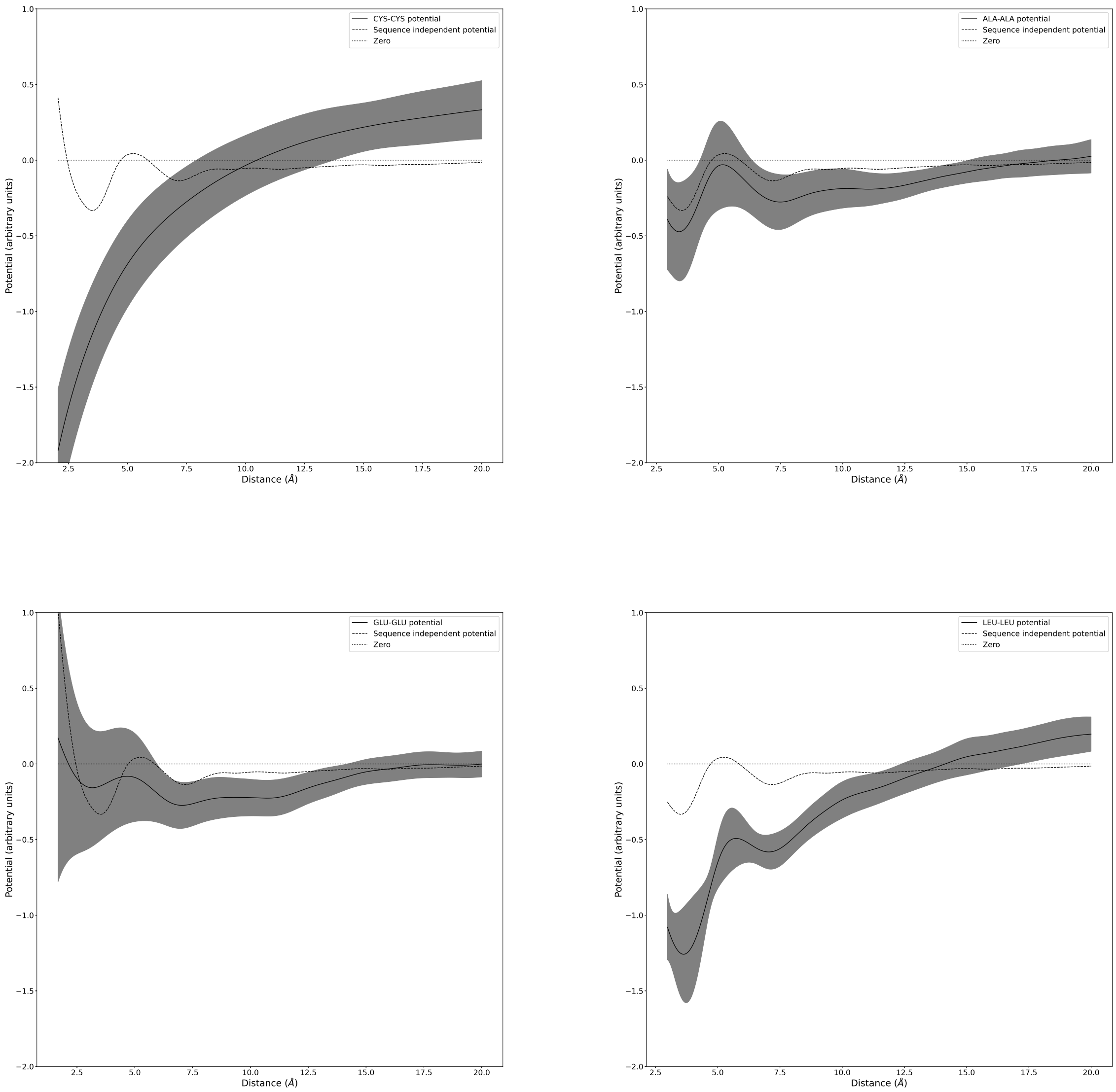

FIG. S10. **Sequence dependent effective statistical potential, HV representation.** Sequence dependent effective potential $\overline{V}(R)$ estimated as an average over all sequence separations $30 \leq m \leq 90$ for the HV representation. The Maxwell distribution with varying Kuhn length $b(M)$ is used as the ideal reference state for a given sequence separation m. The corresponding standard deviation is also shown as the grey area. The sequence independent potential (dashed line) is shown as a reference. **Top left panel:** CYS-CYS. **Top right panel:** ALA-ALA. **Bottom left panel:** GLU-GLU. **Bottom right panel:** LEU-LEU.

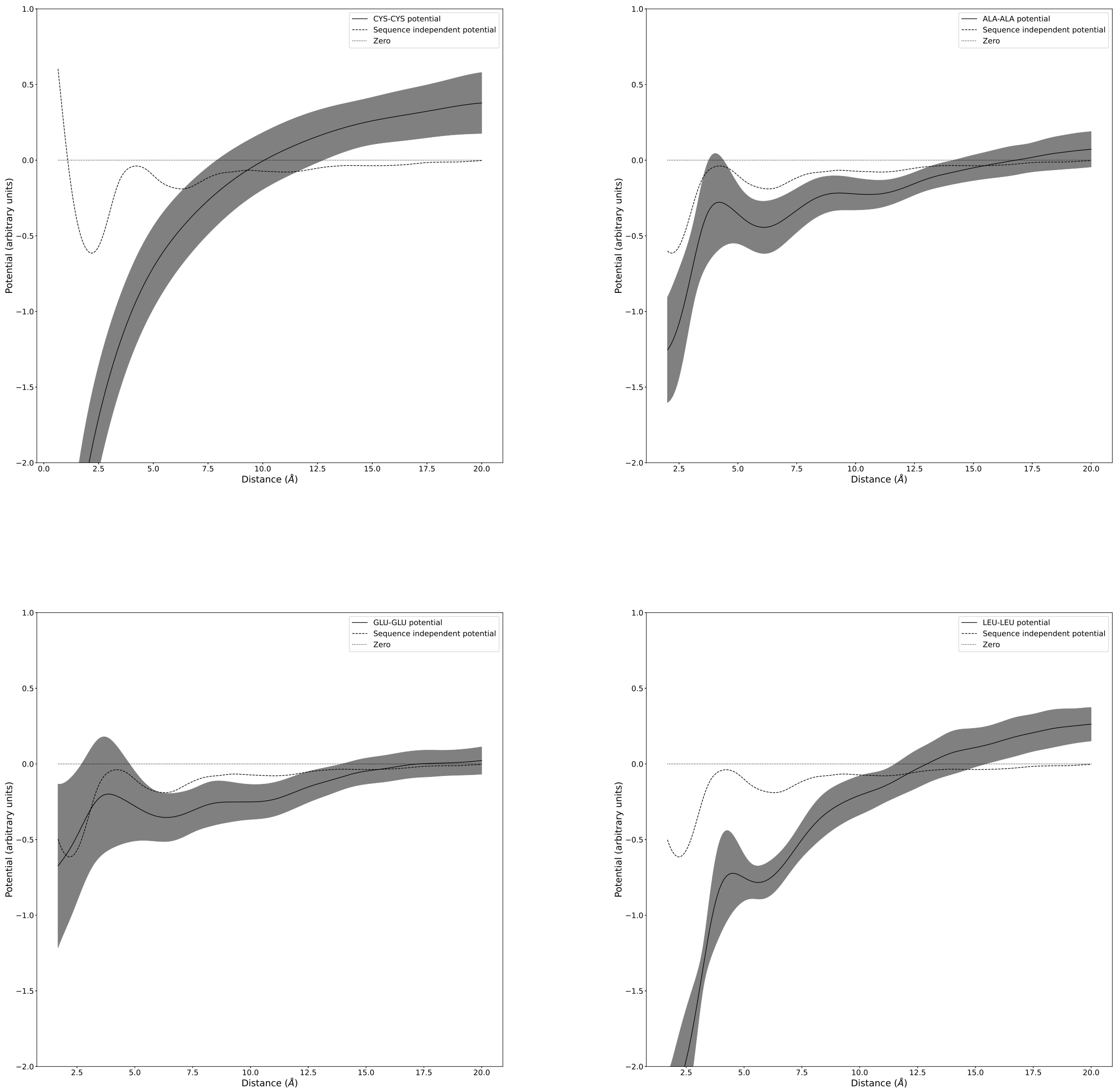

FIG. S11. **Sequence dependent effective statistical potential, HH representation.** Sequence dependent effective potential $\overline{V}(R)$ estimated as an average over all sequence separations $30 \leq m \leq 90$ for the HH representation. The Maxwell distribution with varying Kuhn length $b(M)$ is used as the ideal reference state for a given sequence separation m. The corresponding standard deviation is also shown as the grey area. The sequence independent potential (dashed line) is shown as a reference. **Top left panel:** CYS-CYS. **Top right panel:** ALA-ALA. **Bottom left panel:** GLU-GLU. **Bottom right panel:** LEU-LEU.
