# Supplementary material for: Statistical potentials from the Gaussian scaling behaviour of chain fragments buried within protein globules
Source: PLoS One. 2022 Jan 27;17(1):e0254969. doi: 10.1371/journal.pone.0254969 (PMC8794220; doi:10.1371/journal.pone.0254969)

RESPONSE TO REVIEWERS for the manuscript "Statistical potentials from the Gaussian scaling behaviour of chain fragments buried within protein globules", by S. Zamuner[1], F. Seno[2,3] and A. Trovato[2,3].

1 Institute of Physics, Ecole Polytechnique Fédérale de Lausanne, CH-1015 Lausanne, Switzerland

2 Department of Physics and Astronomy "G. Galilei", University of Padova, Via Marzolo 8, I-35131 Padova, Italy

3 INFN, Sezione di Padova, Via Marzolo 8, I-35131 Padova, Italy

We thank both reviewers for their supportive and careful comments which helped us to improve our manuscript. Below a detailed point-by-point response to their comments.

Reviewer #1: The study finds that long enough buried protein fragments follow Gaussian statistics, and further uses this Gaussian distribution as a reference state in the derivation of statistical potentials. This is an interesting and statistical mechanically rigorous way to define the reference state for statistical potentials. Overall, the manuscript is clearly written and the conclusion is supported by their data.

We thank the reviewer for her/his positive evaluation.

I have a couple of general comments:

1. The Gaussian reference state is m-dependent. Would it be possible to define m-independent Gaussian reference states, at least for certain rang of m values?

We thank the reviewer for her/his observation. In fact, we realized that the description of how we define the "average" potential $\overline{V}(R)$ was possibly misleading and we corrected it in the revised version. As a matter of fact, we always compute $\overline{V}(R)$ as an average of potentials defined for different values of m, each having a different Gaussian reference state (i.e. a different Kuhn length b(m)). In the Flory regime ($70 \leq m \leq 90$) we could in fact use the same Gaussian reference state (with the "uniform" Kuhn length b^* that we estimate in our manuscript) for all the potentials, as suggested by the referee. However, the resulting average potential would in practice be the same as the one computed with m-dependent reference states (see the following figures, where both average potentials are shown for all coarse-graining levels that we used in our work and are superposed one on the other). Finally, we realized that the Flory range was mistakenly labeled as $70 < m < 90$ in the former version of the manuscript. We corrected this to $70 \leq m \leq 90$ (and similarly to $30 \leq m \leq 90$ for the overall range of fragment lengths) in the revised version.

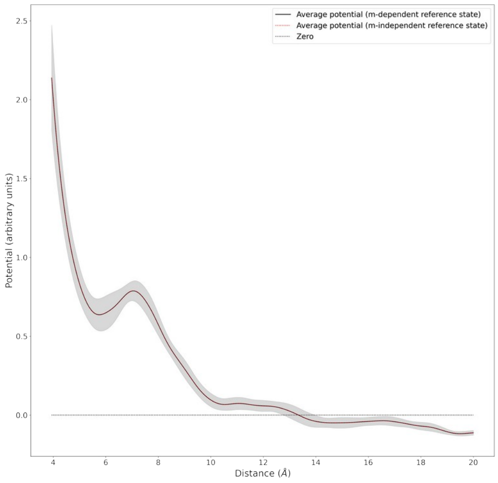

CA coarse-graining

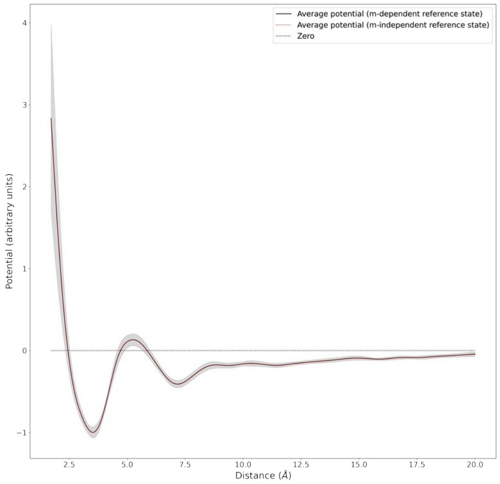

HV coarse-graining

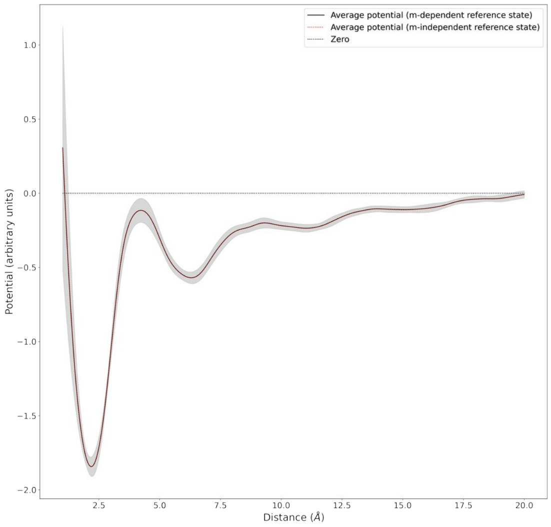

HH coarse-graining

2. For the m region whereby proteins behave like a random Gaussian chain, the use of a Gaussian reference state seems reasonable. What about the regions with small m values (m < 30) or large m values (m>90)?

We thank the reviewer for her/his observation. It is definitely worth discussing why we could not find a Gaussian reference state outside the 30 ≤ m ≤ 90 range. First, we acknowledge that both bounds for the larger range are only approximately determined in our work. The lower bound is necessary to avoid the local rigidity effects brought about mostly by secondary structure elements. The latter ones are otherwise seen to play a role for m < 30, resulting in a non zero tangent-tangent correlation (see S3 Fig), not consistent with a Gaussian regime. The presence of secondary structure elements is instead

fully compatible with the observation of ideal Gaussian statistics for longer fragments. On the other hand, the upper bound m > 90 is due to the lack of statistics caused by the constraint m < $N^{2/3}$ for buried fragments combined with the available protein lengths in the dataset. Were longer proteins available, we would expect the Gaussian statistics to hold for even longer buried fragments. We inserted a paragraph in the discussion section along the above lines.

Reviewer #2: The manuscript is well explained and interesting. I enjoyed reading it. A particular strength is that the limitations of the theoretical approach are described in detail. Some points for the authors to consider when revising the manuscript

We thank the reviewer for her/his positive evaluation and for her/his observations.

- The abstract states that "Gaussian statistics [...] holds for a wide range of fragment lengths [...]". However, the authors note many times in the manuscript that they found deviations from Gaussian statistics at short spatial scales. The sentence in the Abstract should be modified to reflect more fully the data presented in the manuscript.

We agree with the referee and we modified the Abstract accordingly.

- Uncertainties ("error bars") are not provided for some of the fitted quantities. For example b*=3.67 A (line 242).

We agree with the referee (and the editor) that error bars need to be provided. Accordingly, we updated Fig 1b and S4 Fig by showing the error bars associated to the maximum likelihood estimators b(m). Based on the Fisher information evaluated at b(m), standard deviations were estimated as $b(m)/\sqrt{6n(m)}$, where n(m) is the number of fragments in the dataset for a given m (see Table 1). In details, the variance of b(m) is estimated as the inverse of the Fisher information, the latter being minus the second derivative of the log-likelihood function with respect to the parameter b.

Moreover, we also provided the uncertainty for b^*, estimated as the standard deviation of the 21 b(m) values ($70 \le m \le 90$) whose mean defines b^*. For all coarse-graining levels the b^* uncertainty turns out to be 0.01 Å. Such uncertainties were also represented in the updated Fig 1b and S4 Fig.

A proper explanation of how standard deviations for b(m) and b^* was added in the revised manuscript in the Result section.

- How does the Kuhn length b*=3.67 A compare to the average distance between alpha carbons in proteins?

We thank the reviewer for her/his observation. The Kuhn length is in fact close to the average distance, 3.8 Å, found between consecutive CA atoms in protein native structures. We added a sentence in the Result section.

- It is stated i the Discussion that "[...] the use of an ideal chain reference state is well justified **only** for buried protein fragments," (I added * for emphasis). This statement is not backed by the

data presented, the authors have examined only burried fragments and not non-burried/exposed fragments.

We thank the reviewer for her/his observation. We agree that we did not examine the behavior of non-buried/exposed fragments. However, our statement can be backed by the following, admittedly indirect, argument. For fragment lengths $m < N$ but of the same order as chain length N, that is well above the threshold $N^{2/3}$ used here to identify buried fragments, one expects to observe a non Gaussian behaviour characterized by the thermal exponent $\nu = 1/3$ typical of compact globules. In fact, we provide evidence of the “compact globule" scaling of gyration radius with protein length in S2 Fig. We inserted a sentence along the above lines in the Discussion section.

- Variable R is first introduced in line 130, but not defined there. R is
defined later in line 148.

The referee is correct, we introduced the quantity R in a confusing way. First, we now correctly states (lines 116-118 in Dataset subsection) that the gyration radius scaling is used to filter the protein dataset. Second we now introduce R as the end-to-end distance in line 130 (Materials and Methods section) and line 206 (Discussion section).

- Line 173: do the authors mean r.h.s. instead of l.h.s?

Yes we mean r.h.s. Thanks for spotting this!

On behalf of authors,

Prof. Antonio Trovato

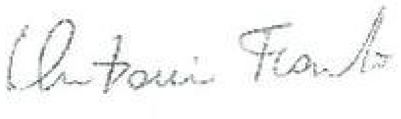

Supplement: Attachment — Submitted filename: Response to Reviewers.pdf [file pone.0254969.s002.pdf]
